# Diptoindonesin G, a new Hsp90 drug

**DOI:** 10.1016/j.jbc.2022.102826

**Published:** 2022-12-23

**Authors:** Anushka Wickramaratne, Sue Wickner

**Affiliations:** Laboratory of Molecular Biology, National Cancer Institute, National Institutes of Health, Bethesda, Maryland, USA

**Keywords:** cancer, chaperones, Hsp90, diptoindonesin G, tanespimycin, inhibitors, Hsp90, heat shock protein 90, ER, estrogen receptor, dip G, diptoindonesin G

## Abstract

Hsp90 is a molecular chaperone that participates in protein folding, activation, and stabilization of substrate proteins. Since many diseases, including cancer, neurodegenerative diseases, and metabolic diseases, are caused by protein misfolding, drugs that inhibit Hsp90 are being pursued as potential targets for treatments. In the recent JBC Editor’s Pick by Donahue *et al*., the authors show that diptoindonesin G is a new Hsp90 inhibitor that promotes degradation of the estrogen receptor, an Hsp90 client, without inducing the heat shock response.

## Commentary

**Figure 1:**
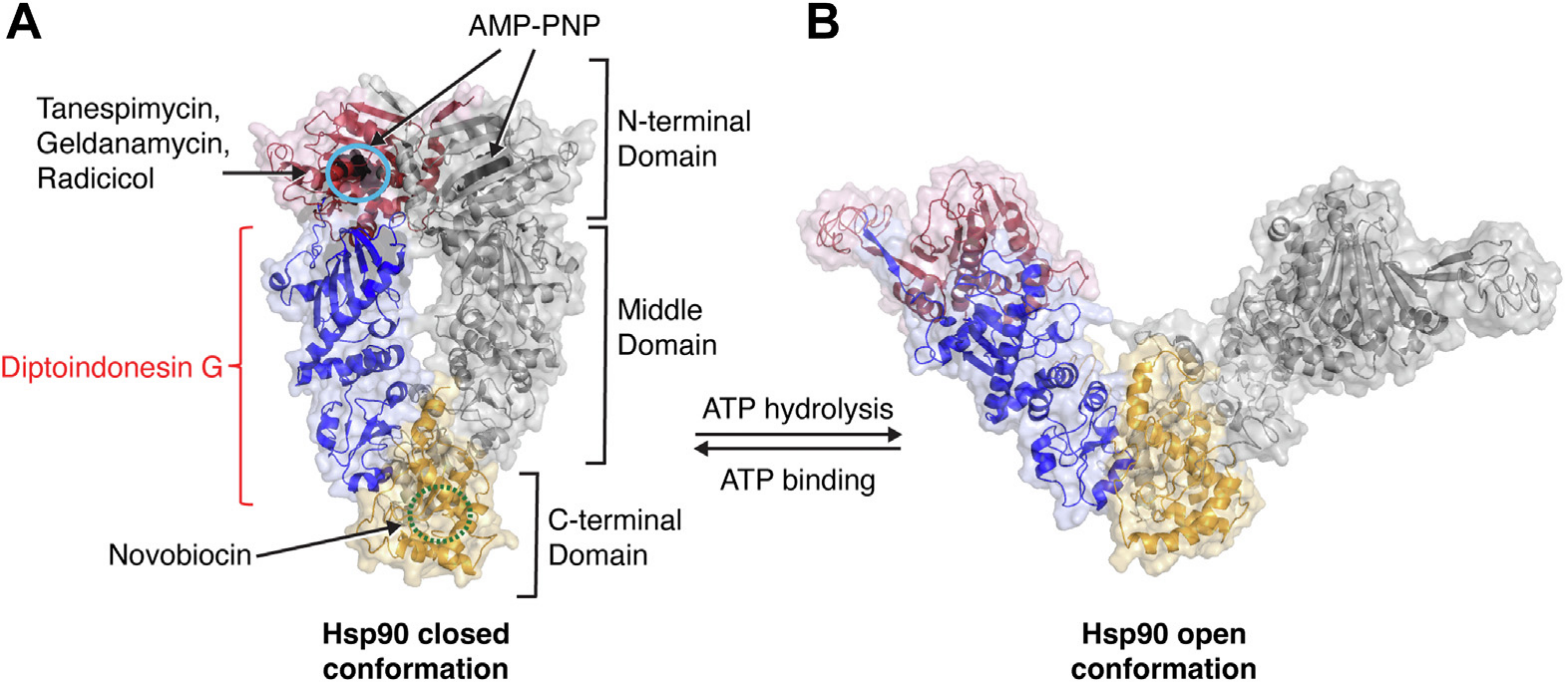
**Diptoindonesin G binding region on Hsp90.***A*, dimer structure of Hsp90 (*Saccharomyces cerevisiae* Hsp82) in the AMP-PNP–bound closed conformation (PDB: 2CG9) showing the N-terminal domain in *rose*, the middle domain in *blue*, and the C-terminal domain in *tan*. The bound nucleotide is shown in *black* as CPK models. The *solid blue circle* indicates the location, as determined by x-ray crystallography, of geldanamycin binding in the Hsp90 nucleotide-binding pocket. The *dashed green circle* represents the putative C-terminal region of Hsp90 where novobiocin and small molecule modulators bind. *B*, dimer model of Hsp90 (*S. cerevisiae* Hsp82) in the open apo conformation and colored as in (*A*). The images were prepared in Pymol. Hsp90, heat shock protein 90.

Hsp90 (heat shock protein 90) is an important and abundant ATP-dependent molecular chaperone, the expression of which is induced by heat shock and other cell stresses (1, 2). It performs essential functions in cellular proteostasis in eukaryotes by remodeling and activating client proteins such as signaling proteins, transcription factors, and regulatory kinases. In addition to promoting protein folding, Hsp90 directs misfolded proteins towards proteasome degradation pathways. Hsp90 functions as a dimer, with each protomer comprising an N-terminal ATP-binding domain, a middle domain, and a C-terminal domain involved in dimerization (Fig. 1). To participate in protein refolding, Hsp90 undergoes large conformational changes that are driven by ATP hydrolysis and modulated by more than 20 Hsp90 co-chaperones as well as the Hsp70 molecular chaperone (1, 2).

Hsp90 has been extensively investigated as a potential target for cancer therapy since it promotes folding and activation of clients, such as the estrogen receptor (ER), that are upregulated in cancers and function as oncoproteins (3, 4). Moreover, Hsp90 in cancerous cells is more active than normal tissues and this results in ‘oncogenic addiction’, where mutated and overexpressed oncoproteins are protected from misfolding and degradation. The first drugs discovered that inhibited Hsp90 belonged to the benzoquinone antibiotic family (3). These inhibitors, including tanespimycin, block protein chaperone activity by competitively binding to the N-terminal ATP-binding site and inhibiting Hsp90 ATPase activity. However, during clinical trials, tanespimycin and its analogs caused dose-limiting toxicity and showed a lack of specificity for individual Hsp90 isoforms and clients (3). They also induced the heat shock response, which led to an upregulation of heat shock proteins, thus protecting cancerous cells from apoptosis and tumor necrosis factor–mediated cell death. C-terminal Hsp90 inhibitors, including novobiocin and its analogs, have been studied as well but also exhibited lack of efficacy and adverse effects in clinical trials (5). Thus, there is currently a need to pursue other approaches to inhibit Hsp90, that is, drugs that bind elsewhere on Hsp90.

In the recent JBC Editor’s Pick by Donahue *et al*. the authors discovered that diptoindonesin G (dip G), an anticancer drug, is a member of a new class of Hsp90 inhibitors (6). Dip G was initially thought to modulate the E3 ligase CHIP (7) and has been studied in ER-positive breast cancer, acute myeloid leukemia, triple negative breast cancer, and prostate cancer. However, the authors now show that, instead of modulating CHIP, dip G is a regulator of Hsp90 function (6). The authors used fluorescence polarization assays to show that a dip G analog, deoxy-dip G, bound Hsp90 with modest affinity (K_d_ ∼ 0.3 μM). Moreover, it had much higher affinity for Hsp90 than for CHIP or ER. They then sought to determine the region of Hsp90 that binds deoxy-dip G. Using Hsp90 protein fragments corresponding to the N-terminal domain, the middle domain, and C-terminal domain, they observed that deoxy-dip G bound to the middle domain with similar affinity as to full-length Hsp90. In comparison, deoxy-dip G exhibited weaker binding to the Hsp90 N- and C-terminal domains. Deoxy-dip G additionally could not be competed off Hsp90 by geldanamycin or radicicol; however, it could be competed off by ATP, leading the authors to posit that induced conformational changes upon ATP binding to the N- or C-terminal domain might cause occlusion of the dip G–binding site in the Hsp90 middle domain. Altogether, their data suggested that, unlike other well-characterized Hsp90 inhibitors, dip G binds to the Hsp90 middle domain (Fig. 1).

The authors further examined the mechanism by which dip G stimulated degradation of ER. They used an ER-positive breast cancer cell line as well as a mutant cell line containing a single knock-in allele of ER to mimic heterozygous expression seen in cancer patients. The ER mutation is a somatic mutation in the ER ligand-binding domain that confers resistance to breast cancer drugs. Using an ER ELISA, they demonstrated that dip G promoted degradation of both WT and mutant ER proteins with similar efficacy. Treatment of hormone-starved ER-positive cells with a proteasome inhibitor and dip G together blocked ER degradation, demonstrating involvement of the proteasome degradation pathway. While tanespimycin and dip G caused comparable degradation of ER in ER-positive cells, proteome-wide analysis of cells treated with either tanespimycin or dip G followed by label-free MS revealed both overlapping and distinct sets of genes whose expression was regulated by the two compounds. Out of 450 proteins regulated by tanespimycin, under 200 were also affected by dip G treatment. Thus, dip G and tanespimycin only regulated a small subset of overlapping proteins, with the majority of proteins being uniquely regulated by one or the other. Donahue *et al*. (6) moreover used RT-qPCR to assess the effect of Hsp90 modulators on the heat shock response in ER-positive cells and demonstrated that, unlike tanespimycin, dip G did not induce the expression of Hsps, including Hsp90, Hsp70, Hsp40, and Hsp27. These results suggest that dip G modulates protein expression in a manner distinct from N-terminal inhibitors.

Thus, the findings presented by Donahue et al. demonstrate that dip G is a member of a new class of Hsp90 inhibitors that bind to the middle domain of Hsp90 and do not affect the stress response. It remains to be determined where dip G binds on the middle domain of Hsp90 and other aspects of its molecular mechanism. Namely, does dip G affect conformational changes of Hsp90, does it affect co-chaperone or client binding, does it interfere with Hsp90-HSF1 interaction, or does it act by some other mechanism? Importantly, it is possible that targeting this middle domain site may lead to the development of new drugs for the treatment of cancer and other protein folding diseases.

## Conflict of interest

The authors declare that they have no conflicts of interest with the contents of this article.
